# The linear chromosome of the plant-pathogenic mycoplasma '*Candidatus *Phytoplasma mali'

**DOI:** 10.1186/1471-2164-9-306

**Published:** 2008-06-26

**Authors:** Michael Kube, Bernd Schneider, Heiner Kuhl, Thomas Dandekar, Katja Heitmann, Alexander M Migdoll, Richard Reinhardt, Erich Seemüller

**Affiliations:** 1 Max Planck Institute for Molecular Genetics, Ihnestr. 63, D-14195 Berlin, Germany; 2 Julius Kuehn Institute, Federal Research Centre for Cultivated Plants, Institute for Plant Protection in Fruit Crops and Viticulture, Schwabenheimer Str. 101, D-69221 Dossenheim, Germany; 3 Biozentrum at the University of Wuerzburg, Institute of Bioinformatics, Am Hubland D-97074 Würzburg, Germany

## Abstract

**Background:**

Phytoplasmas are insect-transmitted, uncultivable bacterial plant pathogens that cause diseases in hundreds of economically important plants. They represent a monophyletic group within the class *Mollicutes *(trivial name mycoplasmas) and are characterized by a small genome with a low GC content, and the lack of a firm cell wall. All mycoplasmas, including strains of '*Candidatus (Ca.) *Phytoplasma asteris' and '*Ca*. P. australiense', examined so far have circular chromosomes, as is the case for almost all walled bacteria.

**Results:**

Our work has shown that '*Ca*. Phytoplasma mali', the causative agent of apple proliferation disease, has a linear chromosome. Linear chromosomes were also identified in the closely related provisional species '*Ca*. P. pyri' and '*Ca*. P. prunorum'. The chromosome of '*Ca*. P. mali' strain AT is 601,943 bp in size and has a GC content of 21.4%. The chromosome is further characterized by large terminal inverted repeats and covalently closed hairpin ends. Analysis of the protein-coding genes revealed that glycolysis, the major energy-yielding pathway supposed for '*Ca*. P. asteris', is incomplete in '*Ca*. P. mali'. Due to the apparent lack of other metabolic pathways present in mycoplasmas, it is proposed that maltose and malate are utilized as carbon and energy sources. However, complete ATP-yielding pathways were not identified. '*Ca*. P. mali' also differs from '*Ca*. P. asteris' by a smaller genome, a lower GC content, a lower number of paralogous genes, fewer insertions of potential mobile DNA elements, and a strongly reduced number of ABC transporters for amino acids. In contrast, '*Ca*. P. mali' has an extended set of genes for homologous recombination, excision repair and SOS response than '*Ca*. P. asteris'.

**Conclusion:**

The small linear chromosome with large terminal inverted repeats and covalently closed hairpin ends, the extremely low GC content and the limited metabolic capabilities reflect unique features of '*Ca*. P. mali', not only within phytoplasmas, but all mycoplasmas. It is expected that the genome information obtained here will contribute to a better understanding of the reduced metabolism of phytoplasmas, their fastidious nutrition requirements that prevented axenic cultivation, and the mechanisms involved in pathogenicity.

## Background

Phytoplasmas are plant pathogens that reside in the phloem, causing a variety of diseases in more than a thousand plant species. They are transmitted from plant to plant by phloem-feeding homopterous insects, mainly leafhoppers (Cicadellidae), planthoppers (Fulgoroidea) and psyllids (Psyllidae) [1]. Phytoplasmas were recently assigned to the novel provisional genus *Candidatus *Phytoplasma [2]. They represent a monophyletic group within the class *Mollicutes *(trivial name mycoplasmas), which has evolved from Gram-positive bacteria [3]. Mycoplasmas are among the smallest self-replicating organisms known, and are characterized by a small genome with a low G+C content, and a lack of a firm cell wall. The genome sizes of phytoplasmas are estimated to range from 0.53 to 1.35 Mb [4] with a GC content between 24 to 33 mol% [5]. Phytoplasmas have two rRNA operons [6], while most other mycoplasmas have only one. Also, in contrast to most other mycoplasmas, phytoplasmas have resisted all attempts of cultivation in cell-free media, indicating that they have a different metabolism than other mycoplasmas and/or a greater reliance on their hosts. As a consequence, these pathogens are poorly characterized on a physiological and biochemical basis.

Molecular phytoplasma research is hindered by difficulties in obtaining high quality DNA from infected plants, and the instability of AT-rich DNA in large-insert genomic libraries. For these reasons, genomes of only four phytoplasma strains have been completely sequenced to date. These phytoplasmas include strains OY-M [7] and AY-WB [8] of '*Candidatus *Phytoplasma asteris' (aster yellows [AY] phytoplasmas) and an Australian and a New Zealand strain of '*Ca*. P. australiense' [9,10]. '*Ca*. P. asteris' and '*Ca*. P. australiense' are closely related and positioned in the same major branch of the phytoplasma clade [1]. In addition, large proportions of the accessible chromosome sequences of the two '*Ca*. P. asteris' strains are syntenic and show high overall DNA homology. All four sequenced phytoplasma chromosomes are circular, like those of all cultivable mycoplasmas examined thus far.

'*Ca*. P. mali' is the causative agent of apple proliferation (AP), one of the most economically important phytoplasma diseases in Europe, severely impairing fruit quality and productivity of the trees. Along with '*Ca*. P. pyri' and '*Ca*. P. prunorum', the causative agents of pear decline and European stone fruit yellows, respectively, and a few other phytoplasmas, '*Ca*. P. mali' forms a second distinct major subclade [11]. In previous work, physical mapping suggested that the chromosomes of '*Ca*. P. mali' strain AT and of '*Ca*. P. prunorum' strain are circular [12,13]. However, in this paper we show that both the two phytoplasmas have linear chromosomes. A similar story has been traced for *Streptomyces *spp., where a circular genetic map was initially constructed until linearity of the chromosomes was finally confirmed [14,15]. Linear chromosomes are rare in bacteria and have only been identified in *Streptomyces *spp., other actinomycetes, the genera *Borrelia *and *Coxiella *and a biovar of *Agrobacterium tumefaciens *[16]. Here we report that not only '*Ca*. P. mali' and '*Ca*. P. prunorum' have a linear chromosome, but also '*Ca*. P. pyri'. In addition, the structural and metabolic characteristics and other features of the completely sequenced chromosome of '*Ca*. P. mali' are described. Comparison of these data with those of the two '*Ca*. P. asteris' strains, from which genome sequences are available, reveal major differences.

## Results

### Chromosome linearity

**Figure 1:**
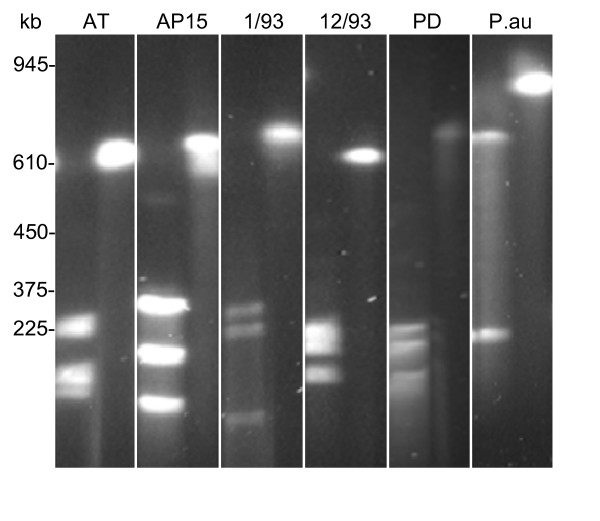
**Linearity of '*Ca*. P. mali' and '*Ca*. P. pyri' chromosomes**. Restriction of linear full-length chromosomes of '*Ca*. P. mali' strains AT, AP15^T^, 1/93 and 12/93 and '*Ca*. P. pyri' (PD) with I-*Ceu*I resulted in three restriction fragments. Two fragments were obtained from the circular chromosome of a '*Ca*. P. australiense' (P.au) strain. The right lane of each PFGE panel shows the undigested chromosome.

Restriction mapping indicated the presence of terminal inverted repeats (TIRs) as known from linear chromosomes of *Streptomyces *species (see below). To prove linearity of the chromosome PFGE-purified, agarose-embedded full-length chromosomes of strain AT were digested with I-*Ceu*I, an intron-encoded endonuclease with a 26-bp recognition sequence that cleaves specifically in the 23S rRNA gene of many bacteria [17]. This digestion resulted in three macrorestriction fragments, as expected for linear chromosomes having two rRNA operons. The same result was obtained with the '*Ca*. P. mali' strains AP15^T^, 1/93, 5/93 and 12/93 and strains of the closely related species '*Ca*. P. pyri' and '*Ca*. P. prunorum' (Figure 1 and data not shown). In contrast to '*Ca*. P. mali' and related phytoplasmas, I-*Ceu*I restriction of the '*Ca*. P. australiense' chromosome revealed only two fragments (Figure 1), as expected for a circular chromosome with two rRNA operons. Similarly, *in silico *digestion of the fully sequenced circular chromosomes of strains OY-M and AY-WB with I-*Ceu*I resulted in two fragments. Furthermore, two fragments were obtained by I-*Ceu*I digestion of the sweet potato little leaf phytoplasma chromosome [18]. Also, *Acholeplasma laidlawii *(accession number CP000896), one of the closest cultivated relatives of the phytoplasmas [3], has a circular chromosome.

**Figure 2:**
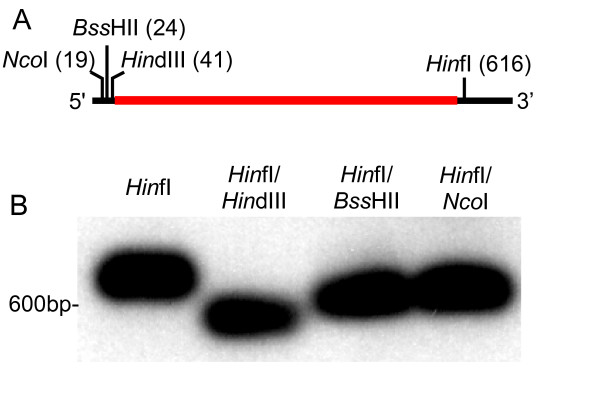
**Completeness of the terminal sequences of strain AT chromosome of '*Ca*. P. mali'**. A: Restriction site prediction of the 5' end of the strain AT chromosome. The PCR generated digoxigenin-labeled probe is displayed in red. B: Southern blot hybridization of strain AT DNA digested with indicated enzymes. The fragment sizes correspond to the restriction sites predicted in the sequence with sizes of 616, 575, 592, and 697 bp for the *Hin*fI (lane 1), *Hin*fI/*Hin*dIII (lane 2), *Hin*fI/*Bss*HII (lane 3), and *Hin*fI/*Nco*I (lane 4) fragments, respectively.

To prove completeness of the AT chromosome sequence, the ends were subjected to restriction analysis using enzymes predicted to cleave near the termini, and were hybridized with a terminal probe to visualize the fragments. In these digestions, fragments were obtained that corresponded in size to the prediction (Figure 2). These results provided evidence that the ends of the chromosome were fully or nearly fully sequenced.

### Terminal inverted repeats in the strain AT chromosome

**Figure 3:**
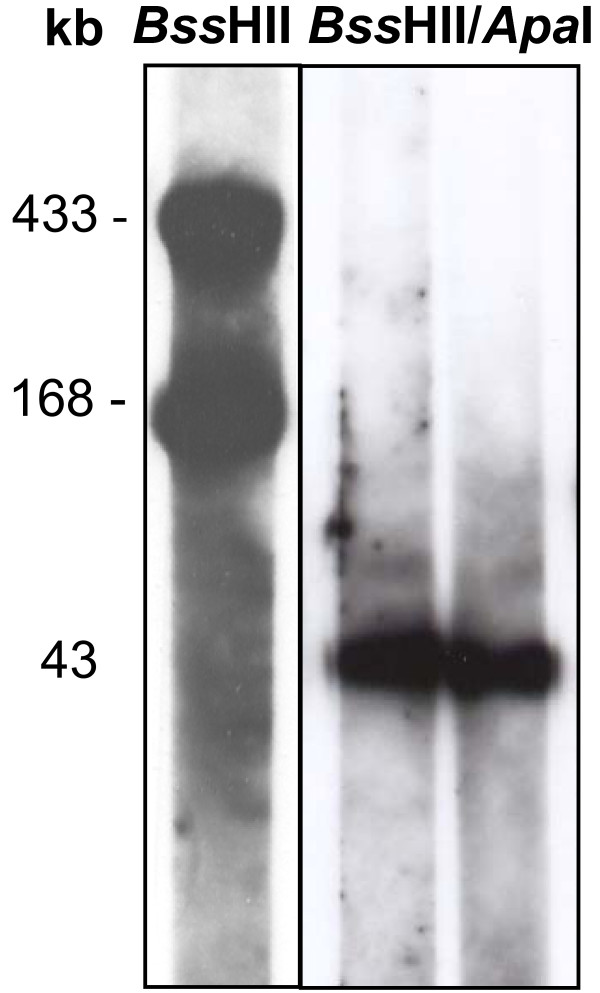
**Southern blot hybridization of restricted and PFGE-separated '*Ca*. P. mali' chromosome with TIR-specific probe znuC**. The two *Bss*HII DNA fragments (left panel) were excised from the gel and separately digested with *Apa*I (right panel). The resulting two distal *Apa*I fragments hybridized equally with the probe.

**Figure 4:**
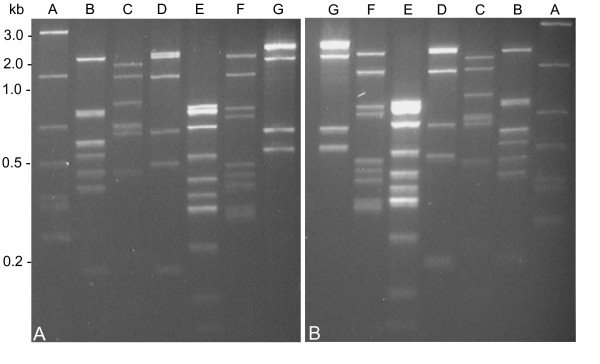
**Identity of the terminal inverted repeats of '*Ca*. P. mali' strain AT**. Identical *Taq*I restriction profiles from seven PCR products were obtained when the terminal 5' (A) and 3' (B) *Apa*I fragments were used as templates. The amplimers cover 97.1% of the *Apa*I fragments. Letters A to G refer to the primer pairs used.

Concomitant restriction mapping during this project using rare-cutting enzymes such as *Apa*I, *Bam*HI, *Mlu*I, *Sma*I and *Xho*I revealed identical profiles at the ends of the chromosome upstream and downstream of the distal *Apa*I sites (data not shown). This suggested the presence of terminal inverted repeats (TIRs), that are typical for linear chromosomes of *Streptomyces *spp. [16]. Evidence on the existence of TIRs was also obtained by separate Southern blot hybridization of the isolated terminal *Apa*I fragments of nearly 43 kb in length with nine PCR-generated probes selected from the 3' *Apa*I fragment. These probes hybridized equally to both the 5' and 3' *Apa*I fragments (see Figure 3 for example). Similarity of the TIRs was also examined by overlapping PCR, employing seven primer pairs covering 41.7 kb of the TIRs. Using each of the PFGE-purified terminal *Apa*I fragments as template, amplimers of the same size were obtained from both fragments. Restriction analysis of the PCR products obtained from each of the *Apa*I fragments with *Taq*I yielded identical DNA profiles (Figure 4).

Sequence identity of the TIRs was confirmed by separate end sequencing of lambda DASH II and FIX II clones specific for the 5' TIR. A total of 110 reads were aligned that perfectly matched to the sequence of both arms, covering 80% of the arm lengths. The length of the perfectly inverted repeats is 42,931 kb. These regions largely coincide with the distal *Apa*I fragments (restriction sites at positions 42,870 and 559,073, respectively). Downstream and upstream of the perfectly inverted region at the 5' end and the 3' end there are transient regions of 650 bp in length consisting of imperfect inverted repeats. At these regions, which differed in 22 positions, several reads aligned. About half of them were identical to the sequence of the right arm and the others were identical to the corresponding region at the 5' terminus (data not shown).

### General genomic features

**Figure 5:**
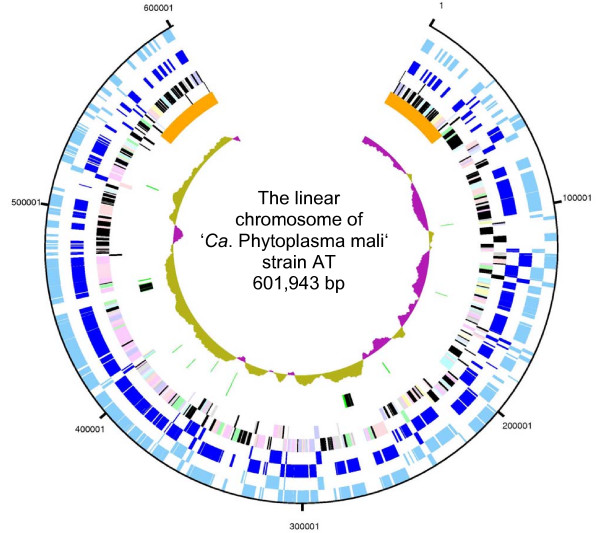
**Genomic organization of the linear chromosome of '*Ca*. P. mali' strain AT**. Circular patterns (from outside): 1 (outer ring), scale in base pairs of the chromosome; 2 (cyan), predicted orthologs to '*Ca*. P. asteris' strain OY-M; 3 (dark blue), predicted orthologs to '*Ca*. P. asteris' strain AY-WB; 4 (multi-colored), predicted coding sequences of strain AT according to COGs; 5 (orange), telomere regions; 6, rrn operons (black), tRNAs (green); 7 (olive and pink), G+C skew. Differences between the phytoplasma chromosomes result mainly from the number of genes with unknown function, integration events and the presence of repair systems.

The linear chromosome of strain AT is composed of 601,943 bp (Figure 5). The chromosome is considerably smaller than that of the AY strains (Table 1) and belongs to the smallest of the bacteria. Position 1 of the sequence is the first nucleotide determined at the 5' end. Extrachromosomal DNA was not identified. The average GC content of the chromosome is 21.4%. This is the lowest value of all mycoplasmas and most walled bacteria analyzed to date. Only very few bacterial genomes, including that of the insect endosymbiont '*Candidatus *Carsonella ruddii', are more AT-rich [19].

**Table 1:** Genome features of '*Ca*. P. mali' strain AT in comparison to '*Ca*. P. asteris' strains AY-WB and OY-M^1^

Strain	AT	AY-WB	OY-M
Chromosome size (kb)	601,943	706,569	860,631
Chromosome organization	Linear	Circular	Circular
G+C content (%)	21.4	26.9	27.7
Protein coding (%)	78.9	73.7	73.0
Coding sequences^2^	497 (16)	671	754 (46)
Average ORF size (bp)	955	776	833
Multiple-copy genes^3^	89	202	250
Genes with assigned function	338 (68%)	349 (52%)	409 (54%)
Conserved hypothetical/predicted novel	72/87	249/73	105/240
Transposases/integrases^4^	1/1	27/1	12/0
rRNA operons	2	2	2
tRNAs	32	31	32
Extrachomosomal DNAs	0	4	2

^1^Chromosome data on strains OY-M an AY-WB from accession number AP006628 and accession number CP000061, respectively.

^2^Annotated pseudogenes in brackets. No information on pseudogenes for strain AY-WB.

^3^Estimation based on BLAST analysis.

^4^Assigned within feature categories note or product.

**Figure 6:**
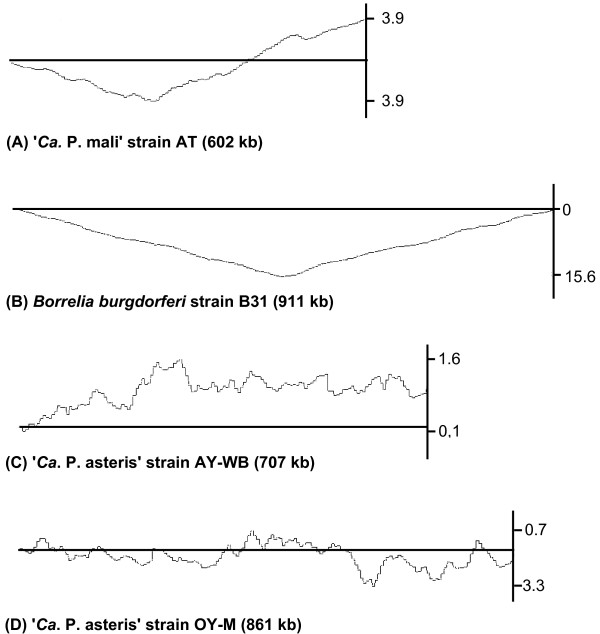
**Cumulative GC skew analyses**. GC skews of the chromosome sequences of '*Ca*. P. mali' strain AT (A), *B. burgdorferi *(B) and the '*Ca*. P. asteris' strains AY-WB (C) and OY-M (D). Graphs show cumulative skews [(C-G)/(G+C)]. Window size 5000 bases. Minima and maxima values are indicated on the axes.

The AT chromosome has a regular cumulative GC-skew pattern that is not impaired by the arms. The pattern is similar to that of the linear chromosome of *Borrelia burgdorferi *(Figure 6A,B). The V-shaped diagram is associated with a characteristic switch in the coding strand preference (Figure 5) and predicts the origin of replication (*oriC*) near position 250,000, just upstream of *dnaAN *and *gyrBA *(positions 250,607 to 257,720). Due to the position of the *oriC *outside the chromosome center, the skew pattern is asymmetric. The predicted position of *oriC *is also supported by oligoskew analyses [20] (data not shown). In contrast to strain AT, the GC-skew patterns of the circular chromosomes of the AY strains are irregular (Figure 6C,D).

**Figure 7:**
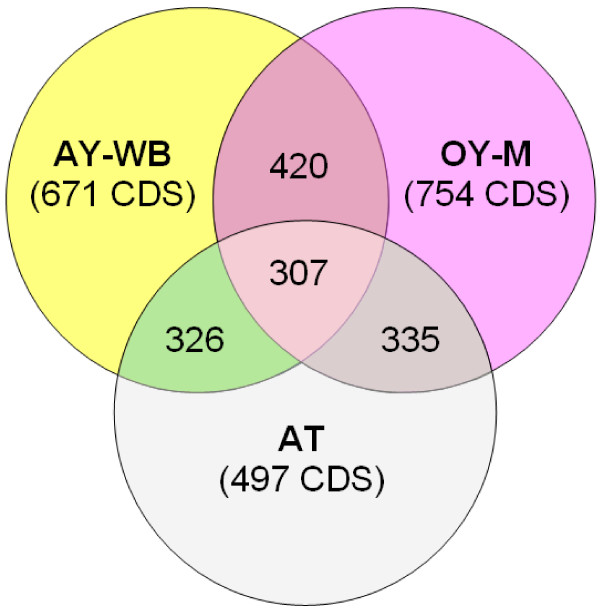
**Deduced shared proteins**. Chromosome-encoded proteins of '*Ca*. P. mali' strain AT were compared with those of '*Ca*. P. asteris' strains OY-M and AY-WB using bi-directional BLASTP. The total number of shared proteins between two or three genomes is indicated. Pseudogenes were integrated with respect to the different annotation style of the genomes.

The AT chromosome consists of a central part or core and large TIRs that occupy 14.3% of the genome. It contains two rRNA operons, 32 tRNA genes and 497 predicted ORFs (Table 1, Figure 5). Sixty-eight percent of the potentially protein-coding genes have assigned functions. In contrast, strains AY-WB and OY-M have 671 and 754 predicted protein-coding genes, respectively, of which only 52 and 54% have assigned functions. Of the non-redundant set of protein-coding genes of strain AT, 62% are shared with the AY strains (Figure 7). The lower number of ORFs in strain AT is not only due to the smaller genome, but also to the lower number of pseudogenes and truncated genes. This results in a larger average size of the genes (Table 1). However, a major reason for the lower number of ORFs in strain AT is the reduced number of multi-copy genes. There are only 89 paralogs in strain AT while 202 and 250 are present in strains AY-WB and OY-M, respectively (Table 1).

There are distinct differences in the coding features between the core and arms. Each arm contains 33 ORFs encoding 19 hypothetical proteins. Eight proteins with assigned function are present in the arms only, and four proteins with assigned function occur in both the arms and core of the chromosome. Three genes were annotated that encode essential proteins occurring in the arms only: they include adenylate kinase (*adk*), transcription termination factor B (*nusB*) and superoxide dismutase (*sodA*). Six of the 12 annotated proteins with predicted functions are related to transport and two to protein degradation. Thus, nearly all annotated genes controlling DNA replication, transcription, translation, cell division, and central metabolism occur in the core. In contrast, genes encoding hypothetical proteins are overrepresented in the arms that also are disproportionately endowed with genes for the import of nutrients from the host and protein degradation. Also, 72% of the paralogs are located in the arms. Similar differences in the coding preferences of core and arms are known from *Streptomyces coelicolor *[21].

In the AY strains the majority of multi-copy genes are organized in clusters of potential mobile units (PMUs). These clusters are composed of genes encoding transposases (*tra5*), primases (*dnaG*), DNA helicases (*dnaB*), thymidilate kinases (*tmk*), Zn-dependent proteases (*hflB*), DNA-binding proteins HU (*himA*), single-stranded DNA-binding proteins (*ssb*) and specialized sigma factors (*sigF*), and a number of genes with unknown function. They are flanked by the transposase and inverted repeats. With this genetic equipment the PMUs are supposed to transpose in a replicative fashion [8]. In strain AT there are only two putative derivatives of PMUs. One of them (positions 477,626 to 505,731) is similar to PMU1 of strain AY-WB but lacks protein HU that is a nonspecific DNA binder and involved in recombination. Also, the flanking *tra5 *gene on one side and the inverted repeats on both sides are missing. This indicates that this PMU is incomplete. The second putative PMU derivative (positions 282,792 to 286,743) is much more reduced and shares only four ORFs with the larger strain AT PMU derivative. There are three other insertions in the AT chromosome that consist of a geminivirus-related element (positions 69,159 to 76,141) and two putative fragments of prophages (positions 216,227 to 222,362 and 328,508 to 342,905).

The low number of paralogs, truncated genes and pseudogenes shows that the genome of strain AT is more condensed than the genomes of the AY strains. The occurrence of incomplete PMUs only may indicate that the AT genome is also less prone to insertion and recombination events. This may be supported by the fact that there is only one transposase gene annotated in the AT chromosome while there are 12 and 27 transposases in strains OY-M and AY-WB, respectively (Table 1). There is also a correlation between the presence of insertion sequences (ISs) and the GC skews. Similar to the different GC skew patterns of strain AT and the AY strains, an irregular skew pattern was identified in the genome of *Mycoplasma mycoides*, 13% of which consists of ISs, whereas *M. mobile *has a regular GC skew and no ISs [22]. Furthermore, the number of PMUs seems to be correlated to the genome size, because strain AT has a smaller genome than the AY strains OY-M and AY-WB. A similar correlation between the frequency of insertion elements and genome size has been found in many other prokaryotes [23]. However, the often obviously truncated PMUs in the AY strains and the low number of PMU derivatives in strain AT, which appear incomplete or severely truncated, indicate that the genomes of phytoplasmas are still undergoing reduction in their size. Thus, expansion or reduction seem to play a major role in phytoplasma genome evolution [8].

### Telomere structures and replication

Bidirectional replication of linear bacterial chromosomes from an internal *oriC *is faced by the problems of copying the 3' ends and protecting the telomeres from enzymatic degradation [24]. Principally, two different modes of replication and protection are known from streptomycetes [25] and borreliae [26]. Of these, the *Streptomyces *mode is characterized by a covalently bound protein complex containing a telomere-protecting terminal protein (Tpg) and a telomere-associated protein that interacts with Tpg and the DNA ends of linear *Streptomyces *replicons [25,27]. This mode is unlikely to apply to strain AT because we could not detect terminal proteins using the proteinase K digestion procedure recommended for this purpose [15]. In addition, the deduced sequences of the two enzymes present in the terminal protein complex, DNA polymerase I (PolA) and topoisomerase I (TopA) [25], have no significant homology to the PolA and TopA proteins of strain AT.

**Figure 8:**
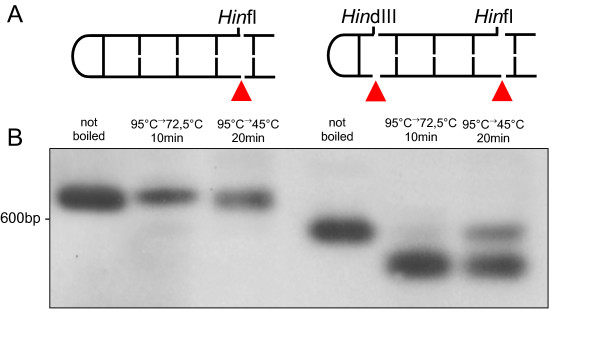
**Renaturation experiments confirming the hairpin structure at strain AT chromosome ends**. A: Graphical display of the terminal ends with position of restriction enzymes *Hin*fI and *Hin*dIII. Red arrowheads indicate cleavage of the termini for subsequent renaturation experiment. B: Single or double digested DNA fragments were either not boiled (lanes 1 and 4) or boiled and slowly cooled down to 72,5°C (lanes 2 and 5) or 45°C (lanes 3 and 6). Digests were separated by agarose gel electrophoresis and hybridized with end-specific probe shown in Figure 2A. Hairpin telomeres (*Hin*fI digest, lanes 2 and 3) renaturated instantly, whereas fragments lacking hairpins (*Hin*fI/*Hin*dIII digest, lanes 5 and 6) renaturated only slowly and partially.

**Figure 9:**
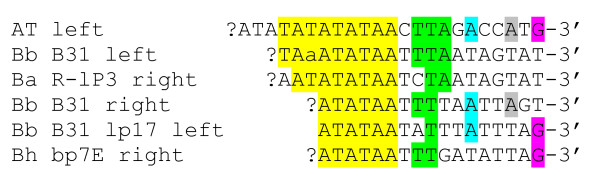
**Similarity of strain AT terminal sequence with hairpin-forming sequences of *Borrelia *spp**. *Borrelia *sequences are from *B. burgdorferi *B31 chromosome (Bb B31), *B. burgdorferi *plasmid lp17 (Bb B31 lp17), chromosome of *B. afzelii *R-lP3 (Ba), and plasmid bp7E of *B. hermsii *(Bh) as shown in [16]. Nucleotides of the strain AT that are found conserved in other sequences are shaded. One position of known variability in the Bb chromosome sequence is indicated by a lower case letter.

In contrast, the AT chromosome has several features indicating that telomere structure and replication principle are similar to those of *Borrelia *species, *A. tumefaciens *and several linear plasmids and phages; consisting of covalently closed hairpin ends and telomere-resolving enzymes [26]. The presence of covalently closed hairpins in strain AT was proven in a renaturation approach similar to that employed to characterize *Borrelia *telomeres [28]. In this experiment the terminal restriction fragments exhibited typical snapback kinetics and renaturated instantly whereas fragments without hairpins renaturated only slowly (Figure 8). The hairpins of strain AT are composed of A and T residues, like those of most or all linear chromosomes and plasmids of *B. burgdorferi *[16,29] (for alignment of terminal sequences see Figure 9). Adjacent to the hairpin-forming termini of strain AT there is a 6-bp palindrome (*Bss*HII site) flanked by short inverted repeats that may form a secondary structure.

The hairpin structure of *B. burgdorferi *and its plasmid lp17 includes the cleavage site of telomere resolvase ResT that shows a cleaving-joining activity by which linear hairpin-forming monomers are created, and telomere duplicates separated to permit chromosome segregation [16,30]. Telomere resolvases are a recently discovered family of DNA breakage and reunion enzymes that are encoded by a few phages, *B. burgdorferi *and a few other organisms. They are functionally related to tyrosine recombinases, cut-and-paste transposases and type IB topoisomerase [31,32]. In strain AT several predicted proteins were identified that showed sequence similarity to ResT and other hairpin-resolving enzymes. However, the overall identity levels were low (12–14%), comparable to those between known resolvases encoded by different linear replicons [32]. Most similar to ResT is hypothetical protein ATP_00103 (identities 13.6%, positives 44%) that has Tyrosine 335 as the active site nucleophile and residue Lysine 224 with known catalytic function at the corresponding positions.

### Energy metabolism and transport

Strain AT has strongly reduced metabolic capabilities due to incomplete or missing pathways. Like the AY strains, many genes coding for amino acid and fatty acid biosynthesis, oxidative phosphorylation, the tricarboxylic acid cycle and the pentose phosphate pathway are lacking. Due to these restrictions it was suggested that phytoplasmas depend on glycolysis for the generation of energy [7]. However, like some other mycoplasmas [33], both strain AT and the AY strains lack the phosphoenolpyruvate-dependent sugar phosphotransferase system (PTS) by which sugars are imported and phosphorylated in order to feed glycolysis. For the import of sugars, only the components of the ABC transporter for maltose (MalKFGE) are present in strain AT, which were also identified in '*Ca*. P. asteris' [8]. In *Thermus thermophilus*, the maltose-binding protein MalE also has affinity to trehalose, sucrose and palatinose [34]. Thus, the maltose transporter seems to be adapted to the nutritional requirement of strain AT as maltose is a major transport sugar in apple phloem [35] and trehalose is a major sugar in the insect hemolymph [36]. However, it is not known if and how these sugars are catabolized because specific maltose- and trehalose-degrading enzymes known from other bacteria [37,38] were not identified.

Strain AT, like the AY strains, possesses the four enzymes (Pgi, PfkA, Fba and TpiA) that are necessary to convert imported phosphorylated sugars into glyceraldehyde-3-phosphate in the upper part of glycolysis (see Additional file 1). However, in contrast to the AY strains, the proteins of the central, energy-yielding part of glycolysis (GapA, Pgk, Pgm, Eno, PykF) are absent in strain AT. The fate of the trioses putatively produced in the preparative glycolysis is unclear. They may be utilized in the glycerophospholipid metabolism. Of this pathway, genes coding for glycerol-3-phosphate dehydrogenase (GpsA), phosphatidate cytidylyltransferase (CdsA), CDP-diacylglycerol-serine O-phosphatidyltransferase (PssA), phosphatidylglycero-phosphate synthase (PgsA), phosphatidylserine decarboxylase (Psd), phospholipid phosphatase (PgpB), and lysophospholipase (PldB) were annotated in the AT genome. These enzymes are involved in the biosynthesis of membrane phospholipids. The same enzymes for phospholipid biosynthesis have been reported to be present in AY phytoplasmas [7,8].

The rudimentary glycolysis identified in strain AT raises the question of how ATP and NADH are generated to provide energy and the redox equivalent. Enzymes required for the utilization of energy sources other than carbohydrates, such as arginine in the arginine dihydrolase pathway that is present in many cultivable mycoplasmas [39], were not found. However, several cultivable mycoplasmas are known to neither metabolize sugars nor arginine, but to oxidize organic acids such as lactate to acetate and CO_2 _[40]. Candidate nutrients for '*Ca*. P. mali' are malate and citrate that can serve as the sole carbon sources for bacteria [41,42] but have not been reported to be utilized by mycoplasmas. Both are the major organic acids transported in apple phloem [35], the plant habitat of the AP agent. Unlike most other mycoplasmas, phytoplasmas have genes coding for symporters of the 2-hydroxycarboxylate (2-HCT) family. These transporters couple the translocation of a solute to the translocation of a co-ion (H^+ ^or Na^+^) in the same direction. Some of the symporters ('exchangers') operate in the exchange mode of transport and couple the uptake of a substrate (e. g. malate and/or citrate) to the export of a metabolic end product (e. g. lactate) [42].

The 2-HCT symporters recognize either malate or citrate as substrates, the exchanger type for both of them [42]. The symporter genes of the AY strains are differently annotated. The proteins deduced from the four genes of strain OY-M were classified as either malate/citrate or Na^+^/citrate symporters [7], and the two genes of the closely related strain AY-WB were annotated to code for Na^+^/malate symporters [8]. The deduced four paralogs of strain AT genes are most closely related to proteins of the AY strains (31–41% identities). Although the strain AT symporters were annotated as malate-specific, a clear classification of their substrate specificity and mode of translocation is not possible without further studies.

Gain of metabolic energy, energy conservation and end product depend on the kind of symporter and the enzymes involved in degradation of malate and citrate. Decarboxylation of the malate or citrate substrate is catalyzed by a protein of the malic enzyme family (e. g. SfcA, MalS, MleS, CitM) that shows specificity to either malate, citrate and/or oxalacetate. In the simplest, proton-donating metabolic system, malate is converted to lactate that subsequently is secreted. In other, more complex pathways, intermediates such as oxalacetate and pyruvate are obtained and NADH, NADPH or ATP are produced [42]. Gain of energy, in particular of ATP, through the catabolism of carboxylic acids by strain AT cannot be evaluated with certainty. It is likely that decarboxylation of malate to pyruvate catalyzed by the NAD^+^-dependent malic enzyme yields NADH. For the conversion of pyruvate to acetyl-CoA the dehydrogenase complex (AcoAB, AceF and Lpd) is present in strain AT. However, the gene encoding phosphotransacetylase (Pta) that catalyzes the conversion of acetyl-CoA to acetyl-P was not identified. This enzyme is commonly found in mycoplasmas [43] and may be among the hypothetical proteins of strain AT. Acetyl-P is converted in an ATP-yielding reaction to acetate by acetate kinase (AckA) [42,44]. This enzyme is present in strain AT and the AY strains.

Strain AT has, similar to the AY strains, five P-type ATPases (4 MgtA, 1 ZntA) that transport a variety of different compounds, including ions and phospholipids, across a membrane using ATP hydrolysis for energy. F-type ATPases (ATP synthases) that use the transmembrane potential for ATP synthesis were neither identified in strain AT nor in '*Ca*. P. asteris' [8]. However, they seem to be present in the cultivable mycoplasmas [43]. As no transport systems for nucleotides and essential nucleic acid precursors were identified in strain AT, it is suggested that there are ATP generation or translocation systems not identified as yet.

In addition to the transporters described above, the AT chromosome contains 39 ORFs that encode transport and binding proteins. These include ATP-binding cassette (ABC) transporter systems for dipeptides/oligopeptides (DppDFCBA, OppA), spermidine/putrescine (PotABCD), cobalt (CbiOQ), Mn/Zn (ZnuBCA), and D-methionine (permease and solute binding components). Also, ABC transporters involved in multidrug resistance (EvbHG) were annotated. This genetic equipment is largely the same as identified in the AY strains [8]. However, amino acid transporter systems other than for D-methionine such as GlnQ and ArtIMPQ that are present in the AY strains are lacking in strain AT. This may indicate that the methionine transport system is not very substrate specific and may also transport other amino acids. A low substrate specificity was also supposed for amino acid transporters of *Mycoplasma pneumoniae *[45].

### Recombination and repair

Strain AT codes for 18 genes involved in recombination and repair including *recAGORU*, *ruvAB*, *ssb *(ATP_00179 and ATP_00398), *gyrAB*, *polA*, *uvrD*, *nfo *(ATP_00027 and ATP_00471), *ungG*, *mutT*, and *hsdS *(fragment). It thus has a rudimentary stock of enzymes that seems to permit homologous recombination, SOS response and excision repair. However, a functioning SOS response has been questioned for mycoplasmas because the central *lexA *gene, which is highly conserved in other bacteria [46], has not been found. With the genes identified strain AT is better endowed than the AY strains that are lacking all *rec *genes as well as *ruvAB *[7,8]. However, *recA *and *recG *are present the peanut witches'-broom agent and the western X-disease phytoplasma, respectively [47,48].

### Pathogenicity determinants

There is no firm data on the mechanisms by which phytoplasmas induce plant diseases. In a recent report it was shown that the severe strain OY-W of '*Ca*. P. asteris' has two sets of some glycolytic genes whereas the mild strain OY-M has only one set. From this finding it was suggested that the difference accounts for the higher virulence of strain OY-W due to a depletion of sugars in the phloem sap and the resulting higher multiplication rate of the phytoplasmas [49]. However, this does not apply for strain AT in which the enzymes of the central part of glycolysis are lacking.

Other possible virulence factors include two putative hemolysins that are encoded by the ORFs *hlyC *and *tlyC*. These genes are also present in the AY strains [8], and do occur in *Xylella *[50] and other walled plant-pathogenic bacteria. With the exception of *secE*, strain AT shares with the AY strains the genes *secA*, *secY*, *dnaJ*, *dnaK*, *yidC*, *ftsY*, *ffh*, *grpE*, *groES *and *groEL *of the protein export and targeting components of the sec-dependent pathway. This indicates the presence of a functional protein translocation system. Hemolysins and protein secretion can be virulence factors by secreting toxins and antimicrobial compounds [51]. Virulence-related proteins were identified in *Streptococcus pyogenes *[52]. Putative protein homologs FtsH (*hflB *gene), an essential ATP-dependent metalloprotease anchored in the cell membrane, are encoded at 11 different loci in the strain AT chromosome. Seven copies have been identified in the AY phytoplasmas strains OY and AY-WB, while most other completely sequenced bacteria such as *Escherichia coli *and *Bacillus subtilis *have only one copy [8,53]. Thus, the AP phytoplasma is unique in having such a large number of *hflB *genes. They may function by degrading host proteins for uptake as essential compounds or by degrading proteins produced by the host cell in the host defense reaction. Other proposed pathogenicity factors are nucleases that may produce precursors for the synthesis of nucleic acids. From cultivable mycoplasmas there is evidence that these nucleases also are potential virulence determinants. A Ca^2+^- and Mg^2+^-dependent endonuclease of *Mycoplasma penetrans *showing significant similarity to endonuclease IV of strain AT and the '*Ca*. P asteris' strains was cytotoxic when added to cultured lymphocytic cells [54].

## Conclusion

'*Ca*. P. mali' is an unusual organism that differs in several aspects significantly not only from '*Ca*. P. asteris', but also from all other mycoplasmas. In comparison to the AY strains, strain AT is characterized by a smaller chromosome, lower GC content, lower number of ORFs and paralogs, fewer PMUs and other insertions and only one transporter type for amino acids. It also seems much better equipped for DNA repair and homologous recombination.

However, more striking are the differences found with regard to chromosome structure and metabolic capacities. '*Ca*. P. mali' and related phytoplasmas are the first mycoplasmas and the first members of the division *Firmicutes *that are known to have linear chromosomes. The linear chromosome of strain AT is characterized by large TIRs and covalently closed hairpin ends. This combination is unique within the bacteria in which two kinds of linear chromosomes can be distinguished. In one group, represented by *Streptomyces *spp., the linear chromosomes usually possess long TIRs ranging from ten to several hundred kilobases. The ends of the TIRs are protected by covalently attached proteins [16]. In the second system, represented by *Borrelia *spp., the TIRs are imperfect and very short (e.g. 26 bp) and characterized by covalently closed hairpin loops at the ends [16]. It is unknown whether the particular structure of the strain AT chromosome evolved in either the apple proliferation phytoplasma subclade, in other phytoplasma groups, or in a phytoplasma ancestor. However, *A. laidlawii*, one descendant of the closest putative phytoplasma ancestor, has a circular chromosome, about 1.5 Mb in length. It also remains an enigma why '*Ca*. P. mali' and related phytoplasmas have linear chromosomes. There are arguments for linearity as a promoter of recombination in the free ends [55]. This recombinogenic property seems not to apply for strain AT as the TIRs are identical and apparently integral parts of the compact chromosome.

'*Ca*. P. mali' shares with '*Ca*. P. asteris' a very limited coding capacity. All are lacking many genes coding for the biosynthesis of amino and fatty acids, oxidative phosphorylation, tricarboxylic acid cycle, pentose phosphate pathway, and the phosphotransferase system. Despite the missing PTS, glycolysis is considered to be the major energy-yielding pathway of '*Ca*. P. asteris'. Sugars are supposed to be imported by a yet unknown mechanism [7,8]. However, unlike in other mycoplasmas [33], the central (triose) portion of glycolysis is completely missing in strain AT. Also, alternative energy-yielding pathways present in other mycoplasmas, in which amino acids (mainly arginine), glycerol, fatty acids, or urea are catabolized [33,56], were not identified in strain AT. The same is true for F-type ATPases (ATP synthases). It thus appears that maltose, malate, and perhaps citrate are the major carbon and energy sources for '*Ca*. P. mali' although no complete catabolic pathways were found. For the degradation of maltose a yet unknown metabolic system is assumed to be used.

For a long time it was expected that complete genome sequences will provide information on the nutritional requirements of phytoplasmas. The sequence data of both '*Ca*. P. mali' and '*Ca*. P. asteris' have revealed the restricted coding potential of phytoplasmas and may explain the failure to cultivate these organisms in cell-free media. The data also indicate that, in contrast to most other mycoplasmas, external sterol is probably not required for phytoplasma growth. However, the question about suitable energy sources cannot be answered as long as the fuels consumed are not known with certainty. Further relevant information may be obtained by annotating hypothetical genes and comparative studies on metabolism. Also, sequences of additional phytoplasma genomes would be helpful to elucidate metabolic features common in phytoplasmas.

## Methods

### Sources, isolation and sequencing of DNA

'*Ca*. P. mali' strains AT [57], AP15^T ^[58], 1/93 and 12/93, '*Ca*. P. pyri' and *Ca*. P. prunorum were collected from symptomatic apple, pear and peach trees, respectively, and were transmitted via dodder (*Cuscuta *sp.) bridges first to periwinkle (*Catharanthus roseus*) and subsequently to tobacco (*Nicotiana tabacum *cv. Samsun and *N. occidentalis*). '*Ca*. P. australiense' originates from cottonbush (*Gomphocarpus physocarpus*) and was maintained in periwinkle [9]. Phytoplasma DNA from infected plants was obtained by either extracting lyophilized tissue using a cetyltrimethyl ammonium bromide (CTAB) procedure followed by repeated bisbenzimide-CsCl buoyant density gradient centrifugation [59] or by isolating full-length chromosomes from stem phloem of *N. tabacum *or periwinkle flowers using a pulsed-field gel electrophoresis (PFGE) protocol [9,12]. DNA obtained by both procedures was used to construct whole-genome shotgun libraries with average insert sizes of 1.2 and 2 kb [60]. Sequencing was carried out using ABI3730XL capillary systems (ABI) and resulted in 20-fold coverage. Sequence quality assessment and assembly were performed with a quality of less than 1 error in 100,000 bases using PHRAP [61] and Consed [62]. The annotated sequence of '*Ca*. P. mali' strain AT has been deposited in Genbank/EMBL/DDBJ under accession number CU469464.

### Terminal inverted repeats

To examine linearity, full-length chromosomes were digested with I-*Ceu*I [17] and fragments were separated by PFGE [12]. Terminal *Apa*I macro restriction fragments were isolated by digesting PFGE-resolved full-length chromosomes with *Bss*HII restriction endonuclease. Following PFGE resolution the two fragments obtained were excised from the gel and separately digested with *Apa*I. The two resulting *Apa*I fragments were resolved by another PFGE and were either blotted for Southern hybridization with TIR-specific probes or excised from the gel and used as PCR templates. The following primer pairs were used to separately amplify most of the two arms of the strain AT chromosome:

A, 5'-ACGACTTTCGACAAGCGACT-3'/5'-GTGTTTCTAAGATCTTTGGC-3';

B, 5'-TGTCATGTATTTGATGCGGG-3'/5'-TTTAGAAGGCAATTTAACCG-3';

C, 5'-TGGATAGAAACAAAGACTTC-3'/5'-TCTTGTAATGCGGGCGAAAC-3';

D, 5'-GGAAAAAGGTCTACTTGAAG-3'/5'-AAACCGACACTACCATTACG-3';

E, 5'-CTCAAATGCCGGTTAATGTG-3'/5'-CAATATCTTTTTTAATCCTCC-3';

F, 5'-GATACGATATTGAATCAAG-3'/5'-TTTTTTGCTTAAATATTTG-3';

G, 5'-TGAAGGCTGATGATTTTAAATC-3'/5'-CGTTATTAAGCTTCTCAAAAG-3'.

For specific sequencing of the TIRs, lambda libraries were constructed from PFGE-purified chromosomal DNA using DASH II and FIX II vectors (Stratagene). Plaque lifts were hybridized with a probe consisting of DNA from the 5' *Apa*I fragment obtained by electroelution [63]. Lambda cloning and plaque lifting were performed as recommended by the supplier of the vector. Digoxigenin (DIG) labeled probes were prepared using the PCR DIG Probe Synthesis Kit or the DIG-High Prime Labeling and Detection Kit (both Roche), and plaque lift and Southern blot hybridization were carried out according to the protocol provided by the supplier. For end sequencing of the lambda clones recombinant phage DNA was isolated using the Lambda Mini Kit following the recommendations of the supplier (Qiagen).

### Data analysis

Structural rRNAs and tRNAs were determined using Rfam [64] and tRNAscan-SE [65]. Protein-coding sequences were predicted by Glimmer3 [66] and annotated in HTGA [67]. Deduced proteins were compared with those of strains AY-WB (accession number CP000061), and OY-M (accession number AP006628) of '*Ca*. P. asteris'. Results were parsed through a script to obtain reciprocal BLASTP [68] hits. Orthologous proteins were predicted using the BLASTP algorithm, considering an e-value of 1-e5 as significant. Alignment length was not taken into account due to the high number of truncated genes in the '*Ca*. P. asteris' strains. The number of protein coding multi-copy genes was estimated based on BLASTP analyses comparing the deduced protein set against itself. Hits were filtered by MSPcrunch [69] to exclude protein hits to itself, at a higher e-value than 1-e5 and below an identity of 70%. Cumulative skew analyses were performed with Artemis [70].

## Authors' contributions

BS and ES performed DNA isolation and purification. KH, MK, HK, BS, ES and RR contributed to library construction, template preparation, sequence determination and assembly. MK, AMM, TD, BS and ES participated in annotation. MK, BS, KH, AMM, RR and ES performed sequence analysis. MK, BS, AMM, RR and ES drafted the manuscript. ES was the initiator of this project. All authors read and approved the final manuscript.

## Supplementary Material

Additional File 1 Fig. S1. Carbon metabolism of '*/Ca/*. P. mali' strain AT Click here for file
