# Supplementary material for: The linear chromosome of the plant-pathogenic mycoplasma 'Candidatus Phytoplasma mali'
Source: BMC Genomics. 2008 Jun 26;9:306. doi: 10.1186/1471-2164-9-306 (PMC2459194; doi:10.1186/1471-2164-9-306)
Supplement: Additional File 1 — Fig. S1. Carbon metabolism of '/Ca/. P. mali' strain AT [file 1471-2164-9-306-S1.pdf]

Carbon metabolism of ‘*Ca*. P. mali’ strain AT

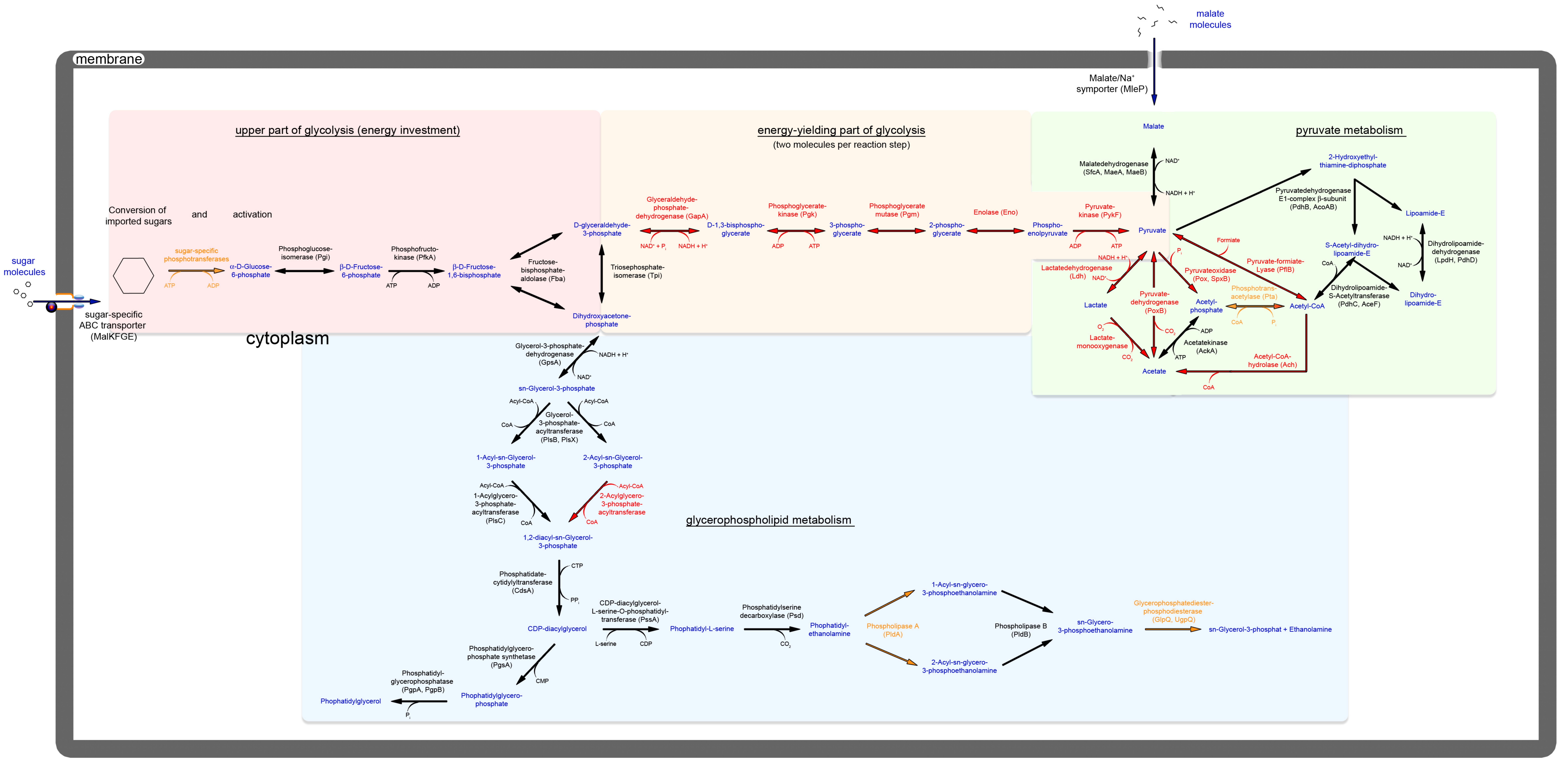

Fig. S1: Key reactions in selected pathways of the carbon metabolism (adapted from KEGG pathway database [http://www.genome.ad.jp/kegg/pathway.html]).
Genes identified in the AT-Genome are shown in black with corresponding black arrows in the pathway while missing genes/pathway steps are marked in red. Products are shown in blue.
Genes or pathway steps, which were not yet identified but could have an equivalent among the hypothetical proteins and would therefore complete a given pathway route,
are highlighted in orange.
